# A method for durian precise fertilization based on improved radial basis neural network algorithm

**DOI:** 10.3389/fpls.2024.1387977

**Published:** 2024-06-05

**Authors:** Ruipeng Tang, Sun Wei, Tang Jianxun, Narendra Kumar Aridas, Mohamad Sofian Abu Talip

**Affiliations:** ^1^ Faculty of Engineering, University of Malaya, Kuala Lumpur, Malaysia; ^2^ Faculty of Electronics and Electrical Engineering, Zhaoqing University, Zhaoqing, Guangdong, China

**Keywords:** durian precise fertilization, durian soil nutrient management, precise nutrient supply, durian planting, durian yield prediction

## Abstract

**Introduction:**

Durian is one of the tropical fruits that requires soil nutrients in its cultivation. It is important to understand the relationship between the content of critical nutrients, such as nitrogen (N), phosphorus (P), and potassium (K) in the soil and durian yield. How to optimize the fertilization plan is also important to the durian planting.

**Methods:**

Thus, this study proposes an Improved Radial Basis Neural Network Algorithm (IM-RBNNA) in the durian precision fertilization. It uses the gray wolf algorithm to optimize the weights and thresholds of the RBNNA algorithm, which can improve the prediction accuracy of the RBNNA algorithm for the soil nutrient content and its relationship with the durian yield. It also collects the soil nutrients and historical yield data to build the IM-RBNNA model and compare with other similar algorithms.

**Results:**

The results show that the IM-RBNNA algorithm is better than the other three algorithms in the average relative error, average absolute error, and coefficient of determination between the predicted and true values of soil N, K, and P fertilizer contents. It also predicts the relationship between soil nutrients and yield, which is closer to the true value.

**Discussion:**

It shows that the IM-RBNNA algorithm can accurately predict the durian soil nutrient content and yield, which is benefited for farmers to make agronomic plans and management strategies. It uses soil nutrient resources efficiently, which reduces the environmental negative impacts. It also ensures that the durian tree can obtain the appropriate amount of nutrients, maximize its growth potential, reduce production costs, and increase yields.

## 1 Introduction

As one of the representatives in tropical fruits, durian is popular for its unique flavor and high nutritional value. The formulation of fertilization strategies is the key issues of agricultural production in durian cultivation. However, the durian fertilization decisions mainly rely on farmers’ experience and traditional agricultural methods, which is subjective and lacks scientific basis. It leads to the effectiveness of fertilization and poses a threat to farmers’ economic benefits and the stability of the supply chain. However, soil properties vary from different regions; traditional fertilization programs fail to consider soil heterogeneity, which leads to unscientific fertilization. It affects the durian growth and quality and negatively impacts land health and sustainability. Therefore, it is important to collect the durian growth data and soil conditions and use relevant algorithms to learn the complex relationship of durian growth for reducing the fertilizer waste and production costs (Zhou et al., 2021; Chanachot et al., 2023).

Precise fertilization decisions can control the input of agricultural production materials and improve the yield and quality of crops. Therefore, some scholars have made some achievements in some crops. Guo et al. (2021) proposed an integrated phenology and climate in rice yields prediction using machine learning methods. It tested 11 phenological, climate, and geographical data and three machine learning methods to predict site-based rice yield, thereby improving the accuracy of rice yield prediction under climate change conditions using integrated machine learning methods. Hossain et al. (Hossain and Siddique, 2020) proposed an online fertilizer recommendation system (OFRS). It analyzed Bangladesh’s national soil database to generate site-specific fertilizer recommendations for selected crops using recommended doses of fertilizer calculated based on soil test values. Kuzman et al. (2021) established a prediction method through an adaptive neuro-fuzzy inference system (ANFIS) to determine the impact of temperature, moisture, humidity, soil type, crop type, nitrogen, potassium, and phosphorus on fertilizer prediction, thereby reducing process costs. Guo et al. (2022) proposed a machine learning-based approach for predicting spad values of maize using multi-spectral images. It used the Mini MCA 6 camera of the drone platform to collect images of corn at different growth stages and established a linear regression model with the spectrum and texture index of different growth stages to accurately monitor the growth and nutritional status of corn for better subsequent fertilization management.

Kanuru et al. (2021) used Global Positioning System (GPS) modules and Internet of Things (IoT) technology to determine the properties of the soil and the types and amounts of pesticides and fertilizers used in effective methods, improving the efficiency of pesticide and fertilizer use to achieve optimal economic benefits. Guo et al. (2023) used hyperspectral images collected by drones, explored multispectral images using the formed dual-band (2D) vegetation index (VI) and 2D texture index (TI), and used five deep learning methods to accurately monitor corn growth, which can help adjust fertilization strategies and achieve precise fertilization. Ahmed et al. (2021) proposed a soil fertilization nutrient recommendation system based on evolutionary calculation. It improves the Genetic Algorithm (IGA) and uses time-series sensor data to make recommendations for various crop nutrient settings. Neighborhood-based strategies were also proposed to handle exploration and exploitation to optimize parameters for maximum yield. Sujatha et al. (2023) proposed a soil fertility classification and fertilization method based on the one-dimensional convolutional neural network. It utilized 1D-CNN to classify soil based on fertility. Classification results were used to specify fertilizers for rice, areca nut, and black/green grams. It also adopted the MinMax normalization and Synthetic Minority Oversampling Technology (SMOTE) to improve the classification efficiency. Lucas et al. (Benedet et al., 2021) used X-ray fluorescence (pXRF) spectrometer to analyze the fertility and element content of 1,975 different soil samples and used a random forest algorithm to establish a spatial distribution model of soil fertility characteristics to achieve soil fertility prediction.

Although the above studies has achieved good results in precise fertilization for some crops, but they are used for other crops and cannot be used for durian because the durian has higher requirements for the growth environment and is quite different from other crops,which is relianced on the information technology infrastructure and the difficulty of equipment maintenance. These methods are mainly the fertilizer effect and nutrient balance. The former has a complex nonlinear relationship between the soil fertilizer amount and multiple soil factors, which does not consider the soil nutrient content. The nutrient balance method needs to keep the dynamic balance, which is corrected. The difficulty of the coefficient is higher. Dong et al. (2020) proposed a method for precise corn fertilization based on wavelet BP neural network, which used wavelet decomposition and reconstruction methods to calculate the yield. However, the combination of wavelet analysis and BP neural network increases the complexity of the model, requiring more computing resources and time for training and verification. Thus, this study proposes an Improved Radial Basis Neural Network Algorithm (IM-RBNNA) in the durian precision fertilization. It extracts and processes the soil sample data and introduces the gray wolf algorithm to improve the Improved Radial Basis Neural Network Algorithm (IM-RBNNA) for calculating the weight ratio, fertilizer amount, and yield of nitrogen, phosphorus, and potassium fertilizers (Song et al., 2020). By comparison, it shows that the IM-RBNNA algorithm can predict the relationship between durian soil nutrient content and yield, which allows durian managers to carry out scientific fertilization based on the prediction results. It reduces fertilizer waste and production costs, achieving sustainability durian planting.

## 2 Algorithms and models design

### 2.1 Radial basis neural network algorithm

**Figure 1:**
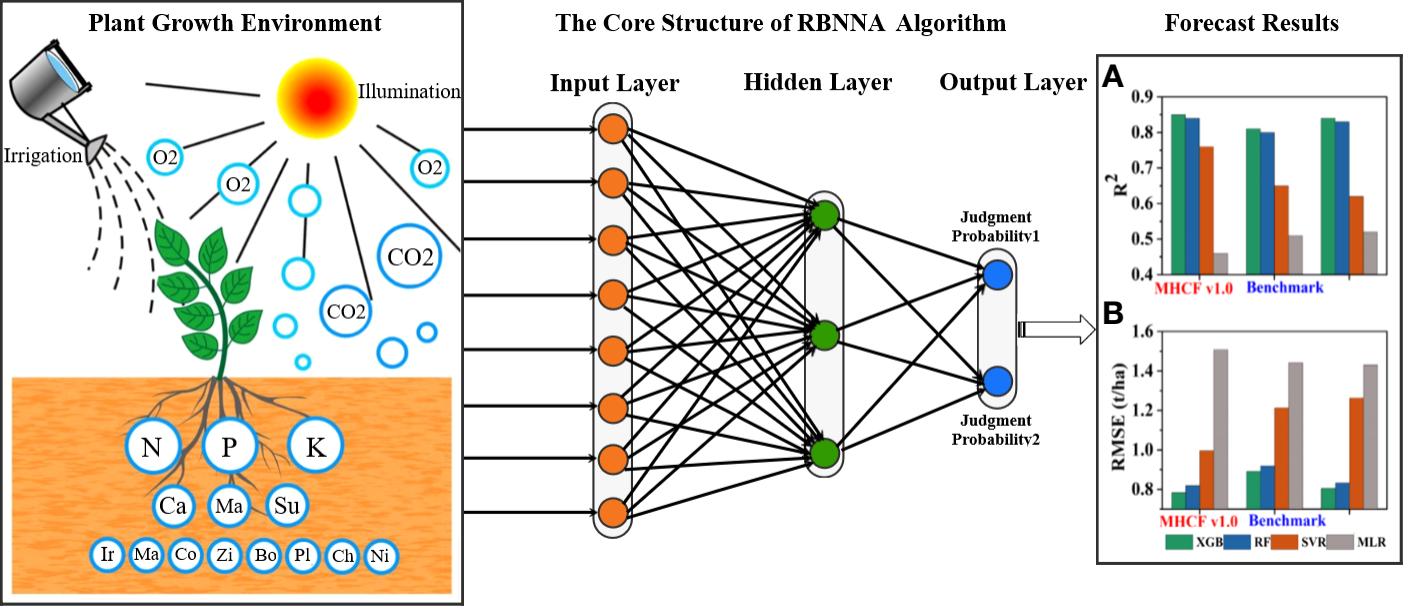
The prediction process of the RBNNA algorithm.


RBNNA is a forward neural network with good performance. It maps nonlinear problems to linear space, transforms them into the adaptive algorithm processing, and maintains the high accuracy and efficiency of the algorithm. RBNNA is a three-layer feedforward neural network consisting of an input, hidden, and output layer (Wang et al., 2023). The input layer is the node that receives the original input data, and each node corresponds to the input feature. The hidden layer is a set of nodes for radial basis functions, which is used to measure the distance between input data and some centers. The output layer produces the final output, which is a linear layer that combines the outputs of the hidden layers. **Figure 1** shows the prediction process of the RBNNA algorithm. The core of RBFNN lies in the radial basis function of the hidden layer, which is a Gaussian or other symmetric function. Gaussian is one of the radial basis functions, which is expressed by Equation 1:

(1) $${\text{G}}_{s}\left(\rho \right)=\text{exp}\left(-{\left|\rho -o_{s}\right|}^{2}/2\sigma_{s}^{2}\right)$$

In Equation 1, ${\text{ G}}_{s}\left(\rho \right)$ represents the output of the s-th basis function, $o_{s}$ represents the center of the basis function, and $\sigma_{s}^{2}$ represents the width parameter.

### 2.2 Gray wolf algorithm

The gray wolf algorithm is a meta-heuristic algorithm proposed by Mirjalili et al (Li et al., 2021), which is derived from gray wolves’ social hierarchy and hunting strategy. In this algorithm, the population is divided into levels A–D. Wolves A control all actions of the wolf pack, which are the supreme leader of the wolf pack. Wolves B assist wolf A in making some decisions, which are some experienced wolves (Xu et al., 2023). Wolves C are responsible for the reconnaissance of the wolf pack, which are responsible for guarding and caring cubs. Wolves D belong to the lowest level of gray wolves and obey the commands of gray wolves from other classes, which are accounting for the vast majority. The best wolves are A, B, and C. They help wolves D to find the favorable area (Verma et al., 2022). First, the wolves need to locate their prey and surround it. The process is shown in Equation 2:


(2) $$\text{Dist}=\left|N\times W_{\varphi }\left(k\right)-W\left(k\right)\right|$$

In Equation 2, D represents the distance between the gray wolf and the prey, N represents the coefficient vector, $W_{\varphi }\left(k\right)$ represents the position vector of the prey, W(k) represents the position vector of the gray wolf, and k represents the number of iterations. The position of k +1 wolves is shown in Equation 3:


(3) $$W\left(k+1\right)=W_{\varphi }\left(k\right)-M\times \text{Dist}$$

In Equation 3, M represents the coefficient vector; other parameters have the same meaning as Equation 2. N represents the calculation process of the coefficient vector sum, which is shown in Equations 4, 5:


(4) $$M=2\omega \times p_{1}-\omega$$

(5) $$N=2p_{2}$$

In Equations 4, 5, ω represents the convergence factor, which decreases linearly from 2 to 0 as k increases; $p_{1}\;$ and $p_{2}$ represent the random number with a value range of (0,1). When the prey is surrounded, the wolves start hunting. The hunting process is carried out under the leadership of wolves A, B, and C. They guide wolves D to track the prey location. The calculation process is as shown in Equations 6–8:


(6) $$A\left(Dist\right)=\left|N_{1}\times W_{a}-W\right|$$

(7) $$B\left(Dist\right)=\left|N_{2}\times W_{b}-W\right|$$

(8) $$C\left(Dist\right)=\left|N_{3}\times W_{c}-W\right|$$

In Equations 6–8, A(Dist) , B(Dist) , and C(Dist) represent the distance between the three wolves and other individuals; $W_{a}$ , $W_{b}$ , and $W_{c}$ represent the current positions of the three wolves; $N_{1}$ , $N_{2}$ , and $N_{3}$ represent the random vectors; and W represents the current position of the gray wolf. The vectors of wolves D in the wolf pack moving toward wolves A, B, and C are represented by $W_{1}$ , $W_{2},$ and $W_{3}$ . The calculation process is as shown in Equations 9–11:


(9) $$W_{1}=W_{a}-M_{1}\times A\left(Dist\right)$$

(10) $$W_{2}=W_{b}-M_{2}\times B\left(Dist\right)$$

(11) $$W_{3}=W_{c}-M_{3}\times C\left(Dist\right)$$

In Equation 9, Equation 10, Equation 11, according to the calculation results of $W_{1}$ , $W_{2}$ , and $W_{3}$ , the final position of wolves D can be determined. The calculation process is shown in Equation 12:


(12) $$D\left(Dist\right)=\left(W_{1}+W_{2}+W_{3}\right)/3$$

**Figure 2:**
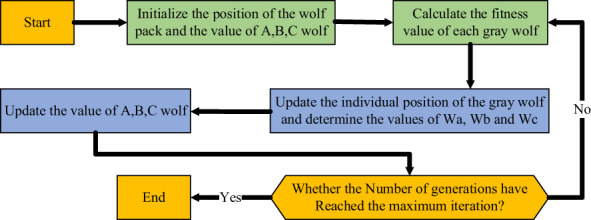
The processing of gray wolf algorithm.

Finally, the hunt is completed by attacking the prey when it cannot move. The processing of gray wolf algorithm is shown in **Figure 2**.

### 2.3 Improved radial basis neural network algorithm

**Figure 3:**
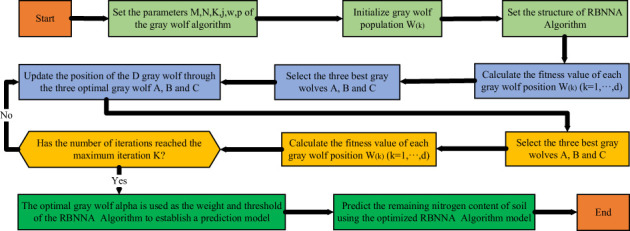
The processing of the gray wolf algorithm for optimizing the RBNNA algorithm.

In order to enhance the predicting accuracy of the RBNNA algorithm, this study proposes an IM-RBNNA algorithm. It uses the gray wolf algorithm to optimize the weights and thresholds of the RBNNA algorithm so that the weights and threshold are optimal. When the output result is different from the expected value, the principle of backpropagation is used to optimize. The threshold and weight of the gray wolf algorithm are used as the weight and threshold of the RBNNA algorithm (Liu and Wang, 2020). The relative error value between the predicted and true value of soil nutrient content is used as the fitness value. The continuous iterative update of the gray wolf algorithm is used to adjust the weights and thresholds of the RBNNA algorithm. The advantages with better global effects can improve the model’s prediction accuracy (Feng et al., 2023). **Figure 3** shows the processing of the gray wolf algorithm for optimizing the RBNNA algorithm.

## 3 Experimental design

### 3.1 Experimental environment

**Figure 4:**
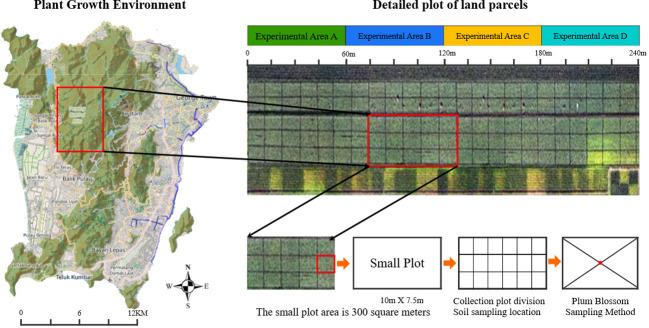
The design of soil sampling locations for this experiment.

This study is conducted in Area 2 of a durian orchard in Penang, Malaysia. It is located in Sungai Pinang Balik Pulau, Penang, which coveres an area of 3,200 acres. The rows of planting density is 5.0 m × 4.0 m. Every acre has 30 plants. The durian trees in this area are all in the peak production period of 15–20 years. During this period, the durian trees have fully developed, so the canopy is dense, which can produce more durian fruits. This area has a tropical rainforest climate, with an average annual temperature of 28°C, an average annual precipitation of 2,525.3 mm, and an annual sunshine count of 2,076.9 h. The study area has significant spatial differences in the growth and yield of durian, which is suitable for the precise fertilization, so this site is chosen to study the precise fertilization. Four experiments are set up in the research area to verify the differences of soil fertility under different algorithms (Portela et al., 2011). From west to east are areas A, B, C, and D, which are used for experiments on different fertilization decision-making methods. Each area is 5 acres. In this plantation soil, the alkaline hydrolyzed nitrogen is 21.5 ± 3.0 mg/kg (low), the available phosphorus is 47.1 ± 0.6 mg/kg (high), the available potassium is 117.7 ± 20.9 mg/kg (low), and the pH is 7.6 ± 0.1 (alkaline). All plots are used for unified measures. **Figure 4** shows the design of soil sampling locations for this experiment.

### 3.2 Data extraction and processing

In order to get the nutrient information of durian soil, five samples are collected within 20 m of the sampling center in each sampling point. The corresponding network of the plot is determined by using manual measurement. The plum blossom sampling method is used in each grid (Zhou and Staver, 2019). Five points of the soil sampling samples are mixed into labeling bags, which have 5,000 sampling points and 150 plots. The sampling time is from February to August 2022. The sample depth is 0–20 cm. These samples are mixed and labeled as soil samples at that point. RTK (real-time kinematic) is used to collect and record the longitude and latitude of the sample point. After the soil samples are naturally air-dried and sieved, the pH is measured to use an electrode method with a water-to-soil volume ratio of 1:1; the organic matter is measured to use the dichromic acid. The potassium method is used to measure the total nitrogen; the copper sulfate digestion method is used to measure the total nitrogen; and the available phosphorus is measured to use the suitable method for neutral and calcareous soils. The soil is measured to use the sodium bicarbonate; the available potassium is measured to use the flame photometry. The fertilizer amount is calculated based on the soil nutrient data, which is obtained from laboratory tests (Liu and Feng, 2017) by using the fertilizer balance model of the target yield method. The physical and chemical properties of the soil are measured through the above laboratory methods to obtain the various nutrient data for each plot (Kim, 2018). **Table 1** shows the nutrient data of some sampling points in the durian orchard.

**Table 1:** The nutrient data of some sampling points in the durian orchard.

Plot	Alkaline hydrolysis nitrogen N (mg/kg)	Available phosphorus P (mg/kg)	Available potassium K (mg/kg)	P application amount (kg/ha)	Amount of N application (kg/ha)	Amount of K application (kg/ha)	Actual output (kg)
2-A1	22.74	5.23	64.1	162.61	76.39	167.05	162.65
2-A2	22.53	6.4	60.8	170.53	82.66	180.97	153.81
2-A3	20.78	5.95	66.17	188.64	79.85	171.99	160.81
2-A4	23.52	5.62	74.65	170.2	86.42	177.48	178.93
2-A5	21.89	8.59	77.06	170.52	72.53	184.26	173.38
2-B1	22.68	5.92	58.27	177.08	73.94	179.42	165.55
2-B2	20.01	7.18	63.35	158.52	80.09	174.23	185.35
2-B3	22.36	6.08	85.2	165.77	88	183.21	157.33
2-B4	24.74	5.92	71.92	161.47	75.21	169.92	163.21
2-B5	24.92	7.64	79.59	182.95	84.26	181.62	182.03

The nutrient contents of alkaline hydrolyzable nitrogen, available phosphorus, and available potassium in the soil vary greatly. For example, the potassium is approximately 200 mg/kg, but the phosphorus is approximately 10 mg/kg. When the cluster analysis is performed, the impact of available phosphorus is almost negligible, which is difficult not to meet the requirements of the soil similarity calculation. In order to solve these problems, this study standardized the data by using the same standard. The normal standardization subtracts each attribute of each data object from the average value of the attribute and then divides it by the variance of the attribute (Mykhailenko et al., 2020). The data standardized by this method reach the standard normal distribution. The data have a mean of 0 and a variance of 1, which is shown in Equation 13.


(13) $$\bar{H_{i}}=\left(H_{i}-R\right)/\alpha_{h}$$

In Equation 13, $\bar{H_{i}}$ represents the i-th standardized data attribute value, $H_{i}$ represents the i-th data attribute value to be standardized, R represents the mean value of the attribute, and $\alpha_{h}$ represents the variance of the attribute. The standardized value of the soil nutrient content is calculated. **Table 2** shows the standardized results of the soil nutrient data in **Table 1**.

**Table 2:** The standardized results of the soil nutrient data in **Table 1**.

Plot	Standardized alkaline hydrolysis nitrogen	Standardized available phosphorus	Standardized available potassium	Standardized application amount	Standardized dosage	Standardized application amount	Standardized measured yield
2-A1	−0.29	−0.77	−0.61	−0.39	−0.33	−0.41	−0.38
2-A2	−0.30	−0.06	−0.83	−0.31	−0.15	−0.24	−0.48
2-A3	−0.52	−0.33	−0.45	−0.10	−0.21	−0.35	−0.24
2-A4	−0.05	−0.55	0.14	−0.32	0.10	−0.14	0.38
2-A5	−0.36	1.37	0.32	−0.31	−0.57	0.06	0.24
2-B1	−0.31	−0.37	−1.17	−0.22	−0.49	−0.08	−0.16
2-B2	−0.72	0.20	−0.69	−0.53	0.08	−0.24	0.58
2-B3	−0.17	−0.23	1.24	−0.38	0.35	0.04	−0.62
2-B4	0.26	−0.37	0.00	−0.47	−0.30	−0.52	−0.36
2-B5	0.29	0.44	0.62	−0.12	0.23	0.25	0.52

### 3.3 Model establishment and evaluation

The Inter@core i7–9700K processor was used in this study, the graphics card is NVIDIA Geforce GTX3080 32GB, the memory is 64GB, the operating system is Ubuntu19.04 64-bit, the deep learning framework is Pyotrch1.9.2, the programming language is Python3.7.1, the integrated development environment is PycharmCE2023, and the drawing tool is Matplotlib 3.1.0. In order to ensure that the data distribution is representative, this study trains 5,000 samples according to the validation set =7:3, which is divided in 3,500 training and 1,500 validation sets. The deep learning network algorithm needs to preset hyperparameters before training, so this study set the hyper parameters to batch after comparison. The number of samples is 6, the epoch is 100, the learning rate optimizer uses the SGD algorithm (Stochastic Gradient Descent) (Thuwajit et al., 2021) to update the weights, the initial learning rate is 0.01, the learning rate decay is 0.001, the activation function uses the Sigmoid function, and the model classifier uses SVM (support vector machine) (Dou et al., 2023).

This study uses the mean relative error (MAPE), the mean absolute error (MAE), and the coefficient of determination ( $R^{2}$ ) to evaluate the performance of the IM-RBNNA and other similar algorithms (Li et al., 2020). The MAPE is used to calculate the relative difference between the actual and predicted values. MAPE is used to calculate the percentage error of each observed value relative to the actual value and then averages it. The smaller the value of MAPE, the better the model’s performance. The MAE is used to calculate the average of the absolute differences between actual and predicted values. The $R^{2}$ measures how well a model fits the data and represents the model’s ability to explain the variation in the dependent variable. In these calculation formula, $U_{j}$ represents the actual value of the soil nitrogen content; $\bar{U_{j}}$ represents the predicted average value of the soil nitrogen content; and U represents the predictive value of the soil nitrogen content. t represents the number of samples. The calculation formula is Equations 14–16, which are as follows:


(14) $$f\left(mape\right)=\frac{1}{t}\sum_{j=1}^{t}\left|\frac{U_{j}-\bar{U_{j}}}{U_{j}}\right|\times 100$$

(15) $$f\left(mae\right)=\frac{1}{t}\sum_{j=1}^{t}\left|U_{j}-\bar{U_{j}}\right|$$

(16) $$f\left(R^{2}\right)=1-\frac{\sum_{j=1}^{t}{\left(U_{j}-\bar{U_{j}}\right)}^{2}}{\sum_{j=1}^{t}{\left(U_{j}-\bar{U}\right)}^{2}}$$

The target yield is the key to durian fertilization recommendations. This study uses the multiple linear stepwise regression methods to determine the durian yield (Sardoei et al., 2023). It determines an initial set containing multiple independent variables and builds a multiple regression formula that does not include this factor. It will run until no more independent variables X can be introduced. The soil nutrient content of the alkaline hydrolyzable nitrogen, available phosphorus, and available potassium are important factors to affect the durian yield, which is relatively in line with the requirements of the above method. The calculation process of the model is shown in Equation 17:


(17) $$Y=e_{0}+e_{1}\times X_{1}+e_{2}\times X_{2}+\ldots +e_{z}\times X_{z}$$

In Equation 17, X represents the independent variable, Y represents the dependent variable, z represents the number of independent variables, and e represents the regression coefficient of each variable. The training of the multiple linear stepwise regression algorithm in this study is conducted in IBMSPSS Statistics 25.

### 3.4 Experimental results

In order to compare the performance of the IM-RBNNA algorithm, this study introduces three methods for comparison: the RBNNA, Backpropagation Neural Network (BPNN) (LI et al., 2019), and Nutrient Balance Calculation Algorithm (NUBCA) (Nannan et al., 2021). The NUBCA algorithm keeps balance between the plants receiving adequate nutrients and their nutrient needs with the available nutrients in the soil. The BPNN algorithm builds the relationship between the plant growth and soil conditions, which uses the backpropagation algorithm for model training and reduces prediction errors by adjusting the weights and biases. This experiment also measures the performance of the four algorithms from three indicators: mean absolute percentage error (MAPE), mean absolute error (MAE), and coefficient of determination( $R^{2}$ ).

#### 3.4.1 The prediction of alkaline hydrolyzable nitrogen in the soil content

**Figure 5:**
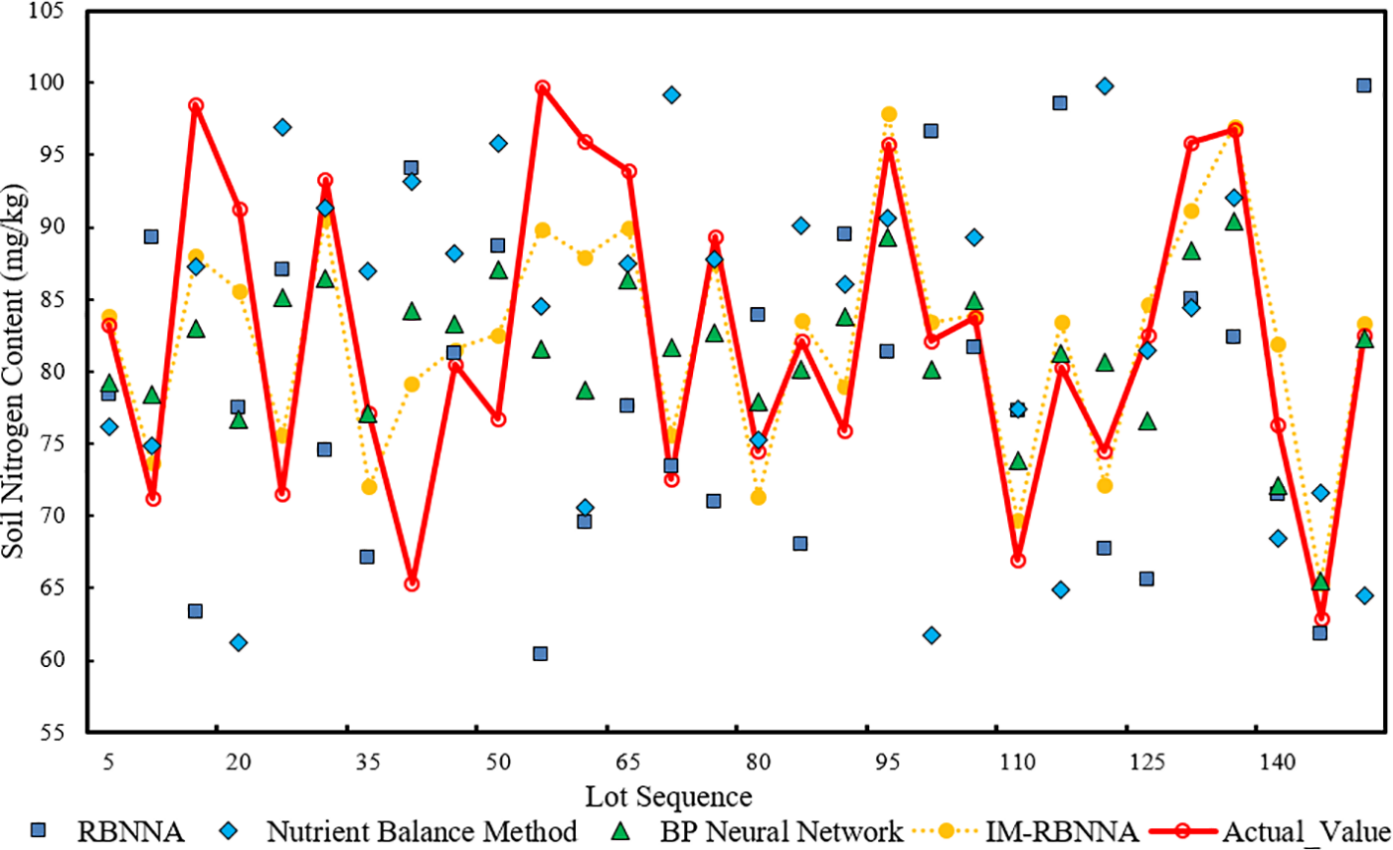
The comparative distribution of predicted alkaline hydrolyzable nitrogen of each algorithm.

**Figure 5** shows the comparative distribution of predicted alkaline hydrolyzable nitrogen of each algorithm. It shows that the maximum, minimum, and average values predicted by the IM-RBNNA algorithm are 97.02 mg/kg, 65.31 mg/kg, and 80.74 mg/kg. The maximum, minimum, and average values in the real soil content are 99.70 mg/kg, 62.93 mg/kg, and 80.50 mg/kg, respectively. **Table 3** shows the performance of different algorithms in predicting soil alkaline hydrolyzable content. By comparing the RBNNA, NUBCA, and BPNN algorithms, the MAPE value of the IM-RBNNA algorithm is 1.61%, which is reduced by 69.41%, 80.26%, and 66.60%; the MAE value of the IM-RBNNA algorithm is 1.403, which is reduced by 57.34%, 76.38%, and 57.99%; and the $R^{2}\;$ value of the IM-RBNNA algorithm is 0.977, which is increased by 8.23%, 28.10%, and 11.17%. It shows that the IM-RBNNA algorithm is more stable than the other three algorithms, which has a smaller fluctuation amplitude, and is closer to the 0-bit horizontal axis. Its prediction effect is better than the other three algorithms, so it can more accurately predict the alkaline hydrolyzable content of durian soil, which is convenient for durian farmers to precise fertilize.

**Table 3:** The performance of different algorithms in predicting soil alkaline hydrolyzable content.

Algorithm name Error index	RBNNA	NUBCA	BPNN	IM-RBNNA
MAPE	5.26%	8.17%	4.82%	1.61%
MAE	3.097	5.94	3.696	1.403
$R^{2}$	0.903	0.762	0.877	0.977

#### 3.4.2 The prediction of available phosphorus in the soil content

**Figure 6:**
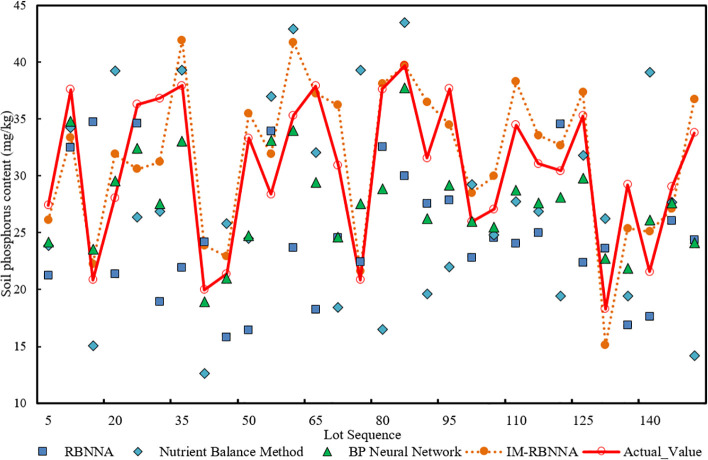
The comparative distribution of predicted available phosphorus of each algorithm.

**Figure 6** shows the comparative distribution of predicted available phosphorus of each algorithm. It shows that the maximum, minimum, and average values predicted by the IM-RBNNA algorithm are 41.93 mg/kg, 15.12 mg/kg, and 29.76 mg/kg. The maximum, minimum, and average values in the real soil content is 39.67 mg/kg, 18.30 mg/kg, and 29.20 mg/kg, respectively. **Table 4** shows the performance of different algorithms in predicting soil available phosphorus content. By comparing the RBNNA, NUBCA, and BPNN algorithms, the MAPE value of the IM-RBNNA algorithm is 10.46%, which is reduced by 35.04%, 47.73%, and 21.66%; the MAE value of the IM-RBNNA algorithm is 3.641, which is reduced by 20.65%, 44.86%, and 24.00%; and the $R^{2}\;$ value of the IM-RBNNA algorithm is 0.835, which is increased by 16.77%, 46.66%, and 18.89%. It shows that the IM-RBNNA algorithm is more stable than the other three algorithms, which has a smaller fluctuation amplitude and is closer to the 0-bit horizontal axis. Its prediction effect is better than the other three algorithms, so it can more accurately predict the available phosphorus content of durian soil, which is convenient for durian farmers to precise fertilize.

**Table 4:** The performance of different algorithms in predicting soil available phosphorus content.

Algorithm name Error index	RBNNA	NUBCA	BPNN	IM-RBNNA
MAPE	16.09%	19.99%	13.36%	10.46%
MAE	5.061	6.306	4.781	3.641
$R^{2}$	0.715	0.572	0.702	0.835

#### 3.4.3 The prediction of available potassium in the soil content

**Figure 7:**
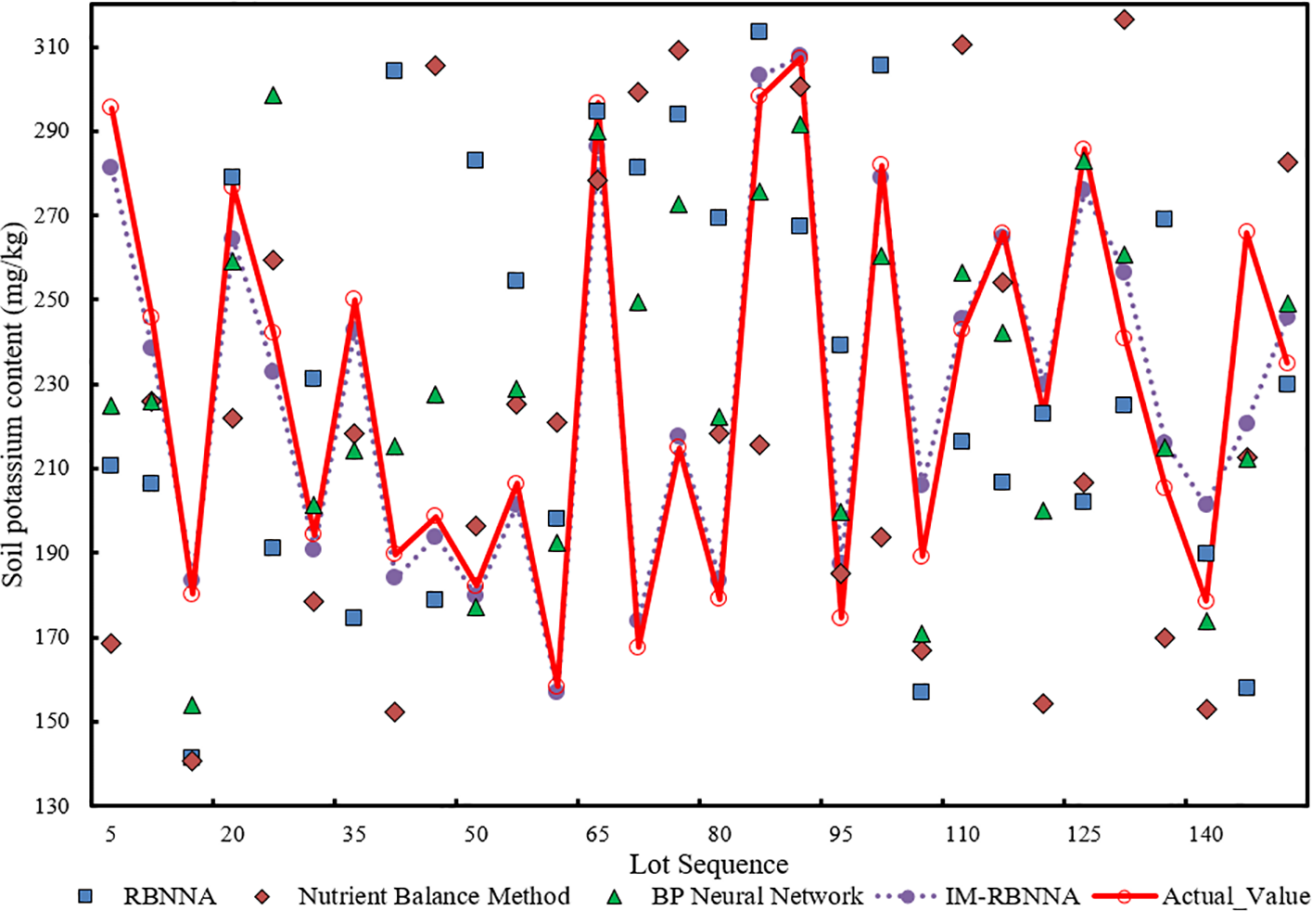
The comparative distribution of predicted available potassium of each algorithm.

**Figure 7** shows the comparative distribution of predicted available potassium of each algorithm.. It shows that the maximum, minimum, and average values predicted by the IM-RBNNA algorithm are 307.73 mg/kg, 157.10 mg/kg, and 228.11 mg/kg. The maximum, minimum, and average values in the real soil content are 307.31 mg/kg, 158.38 mg/kg, and 229.62 mg/kg, respectively. **Table 5** shows the performance of different algorithms in predicting soil available potassium content. By comparing the RBNNA, NUBCA, and BPNN algorithms, the MAPE value of the IM-RBNNA algorithm is 10.46%,which is reduced by 34.95%, 84.36%, and 29.74%; the MAE value of the IM-RBNNA algorithm is 3.641, which is reduced by 13.20%, 21.44%, and 4.20%; and the $R^{2}\;$ value of the IM-RBNNA algorithm is 0.835, which is increased by 8.62%,18.32%, and 4.58%. It shows that the IM-RBNNA algorithm is more stable than the other three algorithms, which has a smaller fluctuation amplitude and is closer to the 0-bit horizontal axis. Its prediction effect is better than the other three algorithms, so it can more accurately predict the available potassium content of durian soil, which is convenient for durian farmers to precise fertilize.

**Table 5:** The performance of different algorithms in predicting soil available potassium content.

Algorithm name Error index	RBNNA	NUBCA	BPNN	IM-RBNNA
MAPE	11.39	15.56	10.95	8.44
MAE	21.01	22.54	19.34	18.56
$R^{2}$	0.838	0.749	0.875	0.917

#### 3.4.4 Prediction of the relationship between soil nutrients and yield

In this study, the multiple linear stepwise regression method determines the fertilizer amount and target yield predicted by four algorithms: RBNNA, NUBCA, BPNN, and IM-RBNNA. The predict time is the annual output of each mature durian tree from 2013 to 2022. The MAPE, MAE, and $R^{2}$ between the four algorithms and the real yield is calculated based on the historical data. In **Figure 7**, the maximum, minimum, and average values predicted by the IM-RBNNA algorithm is 297.75kg/tree, 177.58 kg/tree, and 224.58 kg/tree, respectively. The maximum, minimum, and average values in the real yield is 302.32 kg/tree, 175.87 kg/tree, and 219.21 kg/tree, respectively. **Table 6** shows the performance of different algorithms in predicting the durian yield. By comparing the RBNNA, NUBCA, and BPNN algorithms, the MAPE value of the IM-RBNNA algorithm is 8.28%, which is reduced by 45.09%, 49.13%, and 49.67%; the MAE value of the IM-RBNNA algorithm is 18.56, which is reduced by 41.18%, 43.94%, and 42.22%; and the $R^{2}\;$ value of the IM-RBNNA algorithm is 0.934, which is increased by 14.99%, 21.56%, and 21.08%. It shows that the yield predicted by the IM-RBNNA algorithm based on soil nutrient fertilization is closer to the true value, which helps durian farmers understand the relative contributions of alkali-hydrolyzable nitrogen, available phosphorus, and available potassium to the durian yield. It also makes reasonable decisions based on the prediction results to achieve the goal of maximizing yields.

**Table 6:** The performance of different algorithms in predicting soil potassium content.

Algorithm name Error index	RBNNA	NUBCA	BPNN	IM-RBNNA
MAPE	15.08	16.29	16.44	8.28
MAE	31.56	33.16	32.14	18.56
$R^{2}$	0.812	0.769	0.767	0.934

## 4 Discussion

Although the IM-RBNNA algorithm proposed in this study provides an advanced method for precise durian fertilization, it has some limitations. It is highly dependent on the quality and detail of input data, such as soil nutrient levels and historical yields. Poor data quality or insufficient data volume can significantly reduce the predictive accuracy of the model. However, the comprehensive collection of soil samples and yield data in different growing seasons requires a large cost, so the algorithm needs to be applied for a period of time to gradually improve the accuracy. In addition, the study did not take into account the impact of environmental factors such as soil temperature and humidity, pests, and diseases on durian yield. Take soil moisture as an example; it is an important component of the terrestrial water cycle, which affects the surface material exchange, energy balance, and durian yield (Fang et al., 2020).

**Figure 8:**
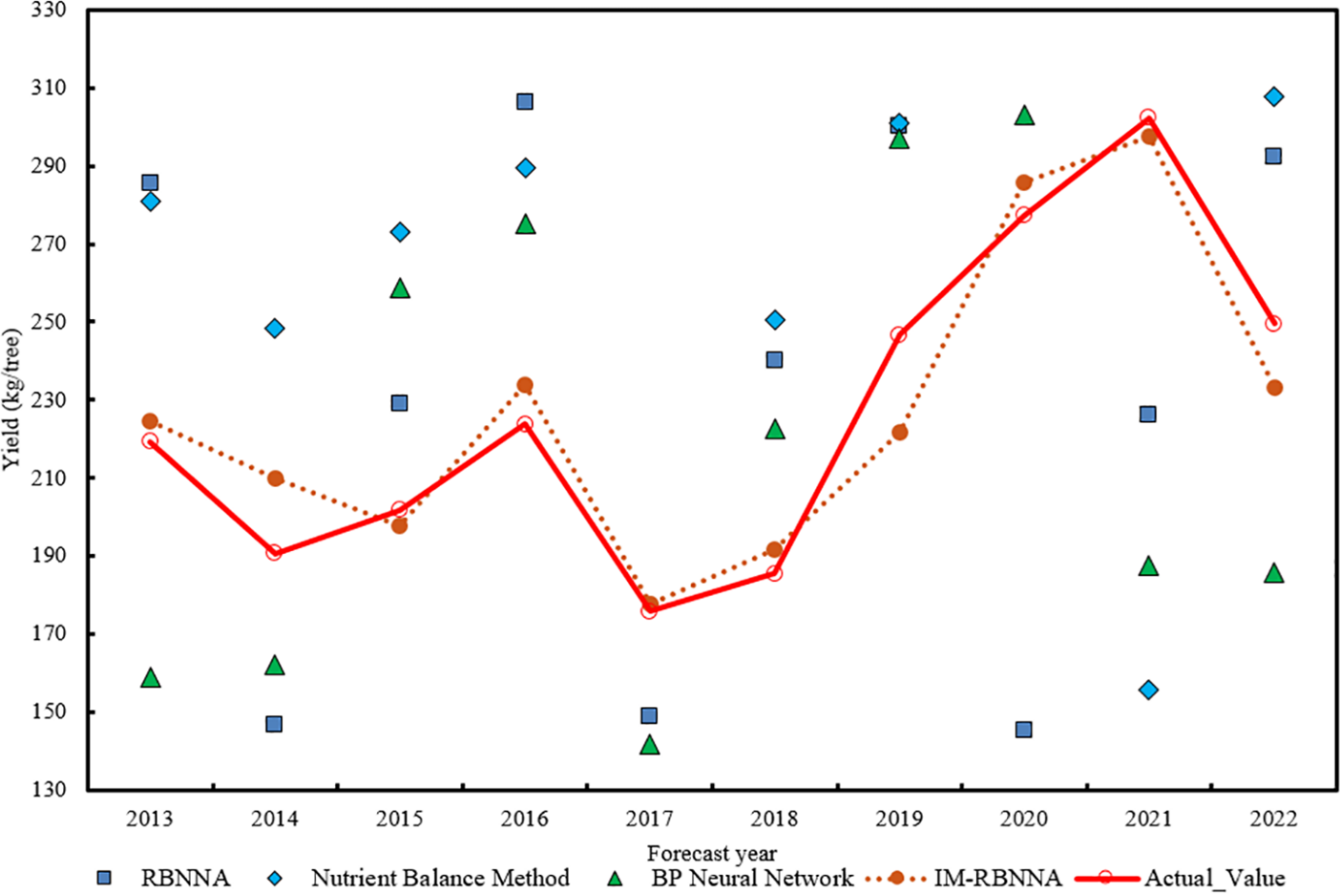
Comparative distribution of predicted values and real values of each algorithm.

**Figure 9:**
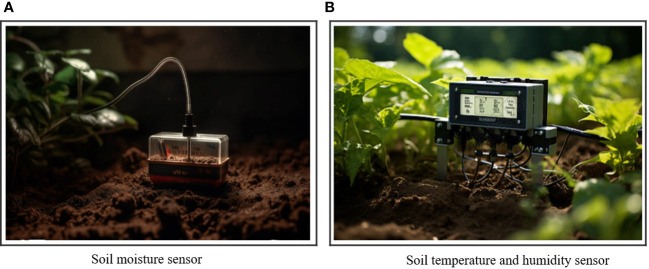
The nutrient and soil temperature and humidity sensor.

In order to improve the yield prediction of the IM-RBNNA algorithm, subsequent studies will collect the soil moisture data and measure it with the TZS-IIW200 soil moisture meter. After setting the sampling points in the laboratory, the field sampling is carried out, and the soil moisture is measured. The latitude and longitude of the sampling points are recorded. The soil moisture data at two different depths of 0–5 cm and 15–20 cm are obtained. The typical slopes of durian topographic undulating sections will be selected. The soil temperature and humidity sensors will be deployed (see **Figure 8**), which obtains soil data at two soil depths of 0–20 cm and 20–30 cm. Two underground plots will be installed. There are 14 sensors, from south to north numbered in sequence. The soil temperature and humidity sensor is TESLA-600. The soil moisture testing accuracy is ± 1%, the soil temperature testing accuracy is ± 0.3°C, and the soil conductivity testing accuracy is ± 2%. The sensor has built the wireless network transmission, which transmits data every hour, works around the day, and records the environmental information in real time. **Figure 9** shows the nutrient and soil temperature and moisture sensors.

In addition, climatic conditions play a vital role in durian production, which includes temperature, humidity, rainfall, and sunlight exposure. For example, temperature is the key factors to the durian growth and fruit development. Warm temperatures is the best environment in for growth durians. The warmer climate aids the flower formation and fruiting process, which increases durian yields (Amran et al., 2023). The proper humidity helps durian plants thrive and enhances pollination, which is crucial for fruit formation. Additionally, durian trees require consistent and evenly distributed rainfall, especially during critical growth stages. The insufficient rainfall causes water stress, which affects the development of flowers and fruits. The excessive rainfall causes waterlogged soil, which affects the root health and nutrient uptake. Finally, sunlight is the key factor affecting photosynthesis. The adequate sunlight is crucial for the healthy growth of durian trees. The insufficient light may weaken the photosynthetic activity, which affects the overall vigor and yield. Through comprehensive training of the above factors and combined with the IM-RBNNA algorithm, the complex relationship between climate conditions and durian yield can be explored, and fertilization strategies can be adjusted according to meteorological changes and soil conditions in different periods. It will help the address climate change, which improves agricultural production capabilities and scientific accuracy of fertilization decisions.

## 5 Conclusions

This study proposes an IM-RBNNA algorithm for the durian precision fertilization. It introduces the gray wolf algorithm to optimize the weights and thresholds of the RBNNA algorithm to enhance the ability to search for optimal solutions and prediction accuracy. It is compared with the RBNNA, NUBCA, and BPNN algorithm. The experimental results show that the IM-RBNNA algorithm is better than the other three algorithms in predicting alkaline hydrolyzable nitrogen, available phosphorus, and available potassium of the soil content. The prediction results between soil nutrients and yield are closer to the true values. The IM-RBNNA algorithm ensures that durian trees obtain the appropriate amount of nutrients and avoid the problem of excess or insufficient nutrients. It helps durian farmers to make the scientific planting plans and management strategies, which can improve the soil fertility utilization. It also reduces production costs and avoids resource waste, which maximizes the growth potential of durian and improves the economic benefits of durian planting.

## Data availability statement

The original contributions presented in the study are included in the article/**Supplementary Material**. Further inquiries can be directed to the corresponding author.

## Author contributions

RT: Writing – review & editing, Writing – original draft, Visualization, Software, Methodology, Investigation, Validation, Formal analysis, Conceptualization, Data curation. SW: Writing – review & editing, Methodology, Validation, Resources, Formal analysis, Conceptualization. TJ: Writing – review & editing, Data curation, Formal analysis, Investigation, Software, Visualization. NA: Writing – review & editing, Project administration, Funding acquisition. MSAT: Writing – review & editing, Supervision, Resources.
