# Supplementary figures and images for: A method for durian precise fertilization based on improved radial basis neural network algorithm
Source: Front Plant Sci. 2024 Jun 5;15:1387977. doi: 10.3389/fpls.2024.1387977 (PMC11188315; doi:10.3389/fpls.2024.1387977)

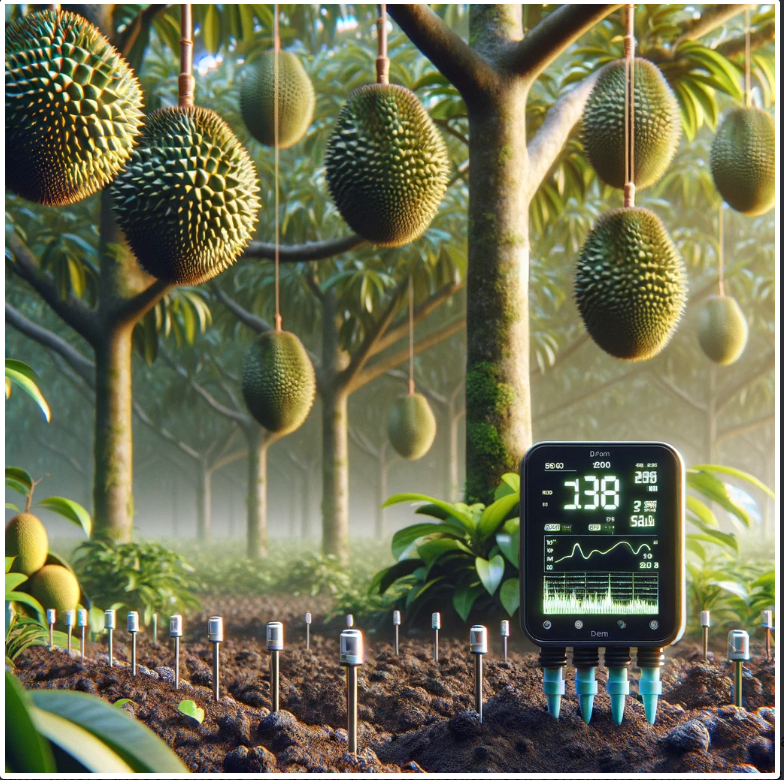

Supplement: Supplementary file 1 [file Image_1.png]
